# Does the Type of Exposure to Workplace Violence Matter to Nurses’ Mental Health?

**DOI:** 10.3390/healthcare9010041

**Published:** 2021-01-05

**Authors:** Farinaz Havaei

**Affiliations:** School of Nursing, The University of British Columbia, Vancouver, BC V6T 2B5, Canada; farinaz.havaei@ubc.ca; Tel.: +1-604-827-4732

**Keywords:** workplace violence, exposure types, intensity, mental health, nursing

## Abstract

Workplace violence is a prevalent phenomenon in healthcare, particularly among nursing professionals. Exposure to workplace violence may be direct through firsthand involvement, indirect through secondhand witnessing, or both. Even though implications for victims of workplace violence have been well-studied, less is known about the various types of exposure and their effects on nurse mental health. The purpose of this study is to examine the impact of workplace-violence exposure types on the mental health of nurses, while accounting for the intensity of the incident/s. This study employs an exploratory correlational design with survey methods. Nurses from British Columbia (BC), Canada, were invited by the provincial nurses’ union to complete an electronic survey in Fall 2019. A total of 2958 responses from direct-care nurses in acute-care settings were analyzed using logistic regression. The results showed that mental-health problems increased with cumulative exposure; even though nurses with solely indirect exposure to workplace violence did not report greater mental-health problems, those experiencing solely direct exposure, or both direct and indirect exposure, were two to four times more likely to report high levels of post-traumatic stress disorder (PTSD), anxiety, depression and burnout compared to their counterparts with no exposure. There is an urgent need for better mental-health support, prevention policies and practices that take into account the type of workplace-violence exposure.

## 1. Introduction

Workplace violence is a prevalent phenomenon in healthcare [1]. Among healthcare workers, nursing professionals are more prone to workplace-violence exposure due to the nature of their work and suboptimal working conditions [2]. International evidence estimated that 25% to 67% of nurses are exposed to at least one type of workplace violence annually. Nurses’ exposure to workplace violence may be direct through firsthand involvement, or indirect when witnessing workplace violence that occurs to their co-workers (also known as secondhand involvement) [3,4]. Even though the antecedents and consequences of direct exposure to workplace violence are well-studied [5,6,7], less is known about indirect exposure types to workplace violence, how the effect of indirect exposure compares to the effect of direct exposure, and whether there is a cumulative effect for those who experience both types of exposure. Examining the effects of workplace violence by exposure type is particularly important given the high prevalence of indirect exposure in healthcare, as many nurses also witness it occur to their colleagues, an experience that may compound the effects of firsthand experience with violence. The purpose of this study is to examine the impact of various exposure types of workplace violence on the mental health of nurses after accounting for the intensity of workplace violence. Gaining a better understanding of exposure types will shed light on more effective prevention policies and interventions tailored to nurses’ needs.

Workplace violence is a complex construct and therefore difficult to operationalize. The majority of workplace-violence research has conceptualized this phenomenon based on its violence types and sources. In a 2003 report from the World Health Organization, workplace violence was classified into two broad categories: physical violence, which was described as the use of physical force leading to physical and psychological harm, and emotional violence, which referred to the use of nonphysical means leading to psychological harm [8]. Since this report, the description of workplace violence has been expanded to encompass other types of violence [9,10,11,12]. For example, a recent Canadian study found that nurses most commonly have direct involvement in emotional abuse (e.g., insults, gestures) (83%), followed by threat of assault (e.g., verbal or written threats intending harm) (78%), physical assault (e.g., bitten, hit, pushed) (67%), verbal sexual harassment (e.g., unwanted intimate remarks of a sexual nature) (55%), and sexual assault (e.g., forcible touching and fondling) (11%) [9]. This body of evidence has also examined workplace violence with respect to its source and consistently found patients and their visitors to be the most common instigators of violence towards nurses, accounting for over 90% of physical and nonphysical workplace violence [9,12].

According to the trauma literature, traumatic events such as workplace violence should also be classified with respect to their exposure type [4,13,14]. Those experiencing workplace violence may be the direct targets of workplace violence themselves (i.e., direct exposure), witness it occur to their colleagues without having direct involvement (i.e., indirect exposure), or encounter both types of exposure [4]. The majority of nursing research has investigated the prevalence of direct exposure to workplace violence, with limited research on indirect and combined direct/indirect exposure [5,6,7]. However, the prevalence of indirect exposure among Canadian nurses has been estimated as 77% [3]. In Australia, as many as 86% of nurses reported being indirectly exposed to at least one type of workplace violence. Despite this evidence, the prevalence of nurses experiencing indirect or combined exposure and its impact on nurses’ health and wellbeing remains unknown.

Workplace violence has also been examined with respect to its antecedents and consequences. Workplace factors such as inadequate staffing, heavy workload and ineffective prevention policies and practices increase the likelihood of exposure to workplace violence and its negative implications for nurses and patients [7,15,16,17]. For example, a study of Australian nurses showed that poorer workplace conditions, such as inadequate RN staffing and dysfunctional relations between co-workers, were associated with more frequent workplace violence exposure [17]. The study also linked workplace violence towards nurses to increased nurse-sensitive patient adverse events such as medication errors and falls [17].

Trauma is an emotional consequence of exposure to events that are perceived as deeply distressing and disturbing [4]. Exposure to traumatic events such as workplace violence puts victims at a greater risk of developing mental-health conditions. The trauma related to mental-health disorders of post-traumatic stress disorder (PTSD), anxiety, depression and burnout are four of the most prevalent and debilitating mental-health conditions worldwide [5]. Among them, PTSD and burnout are the most commonly studied consequences of workplace violence [18,19,20,21], with fewer efforts focused on anxiety and depression [22,23]. Research with nurses has demonstrated similar results, with more intense exposure (operationalized as more frequent exposure to one or more types of workplace violence) having been linked to higher levels in mental-health outcomes including PTSD, burnout, anxiety, depression and insomnia among nurses [6,7,13,22,24,25,26,27].

Workplace violence has other implications for its victims. A systematic review of 68 studies with healthcare workers found that direct exposure to workplace violence is associated not only with negative mental-health outcomes, but also with unfavorable physical, emotional, performance, and economic outcomes [5]. However, within these findings, there is no consensus on the most devastating type of workplace violence. For example, Havaei and MacPhee found that compared to emotional violence, physical violence was a more important predictor of nurses’ psychotropic medication use among Canadian nurses [6]. In contrast, Chang and Cho found verbal abuse and bullying were more strongly associated than physical violence with nurse burnout and other negative nurse outcomes such as job dissatisfaction among Korean nurses [27]. Regardless of this inconsistency, there is a clear consensus across studies that the likelihood of adverse mental-health experiences increases with intense exposure to one or multiple types of workplace violence [26].

While the link between the intensity of workplace violence and health outcomes is well-established, the research examining the relationship between exposure types and health outcomes is limited among nurses. An Australian study examining the association between exposure type and PTSD found that both direct and indirect exposure to workplace violence were related to higher PTSD scores among nurses [13]. A study of approximately 300 Turkish healthcare workers found that both types of exposure were related to increased fears of workplace violence and turnover intent; this study showed that direct exposure had a stronger effect on outcomes than indirect exposure [28]. Neither of these studies, however, investigated the impact of combined direct/indirect exposure, nor did they account for the intensity of workplace violence. This is particularly important as on average over 70% of nurses encounter multiple types of workplace violence from various sources at least once a month [9].

### Theoretical Framework

This study is informed by a theoretical model of trauma that links exposure to traumatic events to the risk of developing mental-health problems, particularly post-traumatic stress symptoms [4]. This model places traumatic events such as workplace violence on a three-dimensional continuum based on their intensity, exposure type and proximity [4,29]. Intensity is conceptualized as the total number of times the same or different types of trauma has been experienced [4]. For example, high-frequency exposure to multiple types of workplace violence is more intense and therefore has more detrimental effects on victims compared to low-frequency exposure to one or more types of violence [30,31,32]. Exposure type reflects direct, indirect or both types of involvement in trauma [4]. The occupational-trauma literature showed that employees who were exposed to workplace violence either directly or indirectly were more likely to demonstrate signs of trauma and mental-health problems compared to their counterparts with no exposure [33,34]. Finally, the proximity of individuals to traumatic events also matters to their risk of being negatively impacted [4,29,35]. In the current study, exposure type also encompasses proximity, as direct exposure to violence implies a closer proximity to trauma compared to indirect exposure [4].

## 2. Materials and Methods

This was a provincial study of nurses in British Columbia (BC), Canada, using a cross-sectional correlational survey design. In September 2019, the provincial nurses’ union sent an email invitation to its members (~48,000) asking them to complete an electronic survey on psychological health and safety in the workplace [3]. Participants were informed of the confidentiality of their responses, the voluntary nature of their participation and that survey completion would indicate informed consent. To increase response rate, several strategies were utilized, including a two-month data collection period, social media advertisement, weekly reminders and a raffle draw. Overall, 5512 surveys were returned, yielding a response rate of 12%. For this study, actively working nurses who identified their role as direct-care provider, reported working in acute care and had complete information on workplace-violence exposure type were included. The final sample consisted of 2958 nurses. Ethics approval was obtained from the University Behavioural Research Ethics Board (approval number: H18-02724).

### 2.1. Measures

There were two key workplace violence questions on the survey. The first question asked nurses to report the frequency of direct exposure to 5 types of workplace violence over the last year, including physical violence, threats of assault, emotional abuse, verbal sexual harassment and sexual assault [9]; response options were on a 7-point scale ranging from never (0) to everyday (6). The second question asked participants whether they had witnessed any type of workplace violence over the last year without being directly involved (0 = no, 1 = yes) [13]. To determine participants’ exposure types, their responses to the first question were dichotomized (0 = never, 1 = ever) and considered in conjunction with their responses to the second question. Possible exposure types included (a) “no exposure”, (b) “only direct exposure”, (c) “only indirect exposure”, and (d) “both direct and indirect exposure”.

Participants’ responses to the first question, on a 7-point scale, were summed to measure the intensity of exposure to 5 types of workplace violence. Sum scores ranged from 0 to 30, with higher scores indicating more-intense exposure [9].

Mental-health outcomes were measured using four previously validated scales. First, the Post-Traumatic Stress Syndrome-14 Question Inventory (PTSS-14) with 14 items rated on a 7-point scale ranging from 1 (never) to 7 (always) was used to measure PTSD [36]. Based on established cutoffs [36], sum scores of 14–45 were recoded to indicate no-to-mild PTSD (0), and sum scores ranging from 46–98 were recoded to represent potential PTSD (1). Second, the General Anxiety Disorder Scale (GAD-7) consisting of 7 questions on a 4-point scale ranging from 0 (not at all) to 3 (nearly every day) was used to measure anxiety; sum scores ranging from 0–9 were recoded to indicate no-to-mild anxiety (0), while scores from 10–21 were recoded to represent moderate-to-severe anxiety (1) [37]. Third, the Patient Health Questionnaire (PHQ-9), composed of 9 items rated on a 4-point scale ranging from 0 (not at all) to 3 (nearly every day), was used to measure depression [38]. Established cutoff scores of 0–9 were used to indicate no-to-mild depression (0) and scores of 10–29 used to reflect moderate-to-severe depression (1) [38]. Finally, burnout was measured using the 22-item Maslach Burnout Inventory-Human Services Survey (MBI-HSS), which is composed of 3 subscales: emotional exhaustion (EE), depersonalization (DP) and personal accomplishment (PA). Items were rated on a 7-point scale ranging from 0 (never) to 6 (every day). Previously established cutoff scores were used to categorize the response data of each subscale: EE scores ranging from 27–54 represented high levels; DP scores ranging from 13–30 represented high levels; PA scores ranging from 0–31 represented low levels [39]. Similar to previous research, high EE and DP, or high EE and low PA were used as indicators of burnout [40].

The survey also included a series of researcher-developed questions on participant demographics. These questions asked about participant age, gender (female, male), professional designation (licensed practical nurses, registered nurses, registered psychiatric nurses) and employment status (full-time, part-time, casual).

### 2.2. Data Analysis

Data were analyzed using descriptive statistics, cross-tabulation, and logistic regression. Crosstab analyses were used to examine the bivariate relationship between exposure type and mental-health outcomes. Logistic regression was used to examine the impact of exposure type on mental-health outcomes over and above the effect of workplace-violence intensity and nurse demographics. As such, four demographic variables and one intensity variable were entered into the first model, followed by three dummy-coded exposure variables in the second model: “only indirect exposure”, “only direct exposure”, and “both indirect and direct exposure”. The Statistical Package for Social Sciences for Windows 25.0 (SPSS Inc., Chicago, IL, USA) was used for data analysis.

## 3. Results

Table 1 shows descriptive statistics on demographics and key study variables. An overwhelming majority of respondents were female and registered nurses (RNs). Nearly two-thirds identified their employment status as full-time as opposed to part-time (25%) or casual (12%). On average, respondents were 39 years old and reported a mean score of 6.8 (SD = 5.5) on violence intensity, which was more predominantly driven by nurses’ experiences of emotional abuse (86%), threat of assault (83%) and physical assault (74%), compared to verbal sexual harassment (59%) and sexual assault (13%). Of note is that nearly 85% of our sample reported experiencing more than one type of workplace violence over the last year. The two most commonly reported exposure types were “both direct and indirect exposure” (75%) followed by “only direct exposure” (19%). A smaller group of respondents reported no exposure (5%) followed by “only indirect exposure” (2%). One out of two nurses met the criteria for PTSD, and nearly one out of three nurses met the criteria for anxiety, depression and burnout.

Table 2 shows the crosstab for workplace violence exposure types by mental-health outcomes. There were significant differences in the proportion of mental-health problems across different exposure groups (*X*^2^ = 64.33–157.09, *p* < 0.001). While smaller proportions of nurses with no exposure met the criteria for PTSD, anxiety, depression and burnout (Range: 5–15%), greater proportions of those with “both direct and indirect exposure” reported these unfavorable mental-health outcomes (Range: 31–56%). Nurses in the “only direct exposure” group had greater mental-health problems (Range: 22–37%) than their peers with “only indirect exposure” (Range: 12–17%), but fewer mental-health problems than the “both direct and indirect exposure” group.

Table 3 shows the logistic regression results. The type of exposure to workplace violence was significantly related to mental-health outcomes after controlling for nurse demographics and workplace-violence intensity. Nurses that reported “only direct exposure” and “both direct and indirect exposure” were two to four times more likely to meet the criteria for PTSD (*OR* = 2.18–3.11, *p* < 0.01), anxiety (*OR* = 3.49–3.85, *p* < 0.01), depression (*OR* = 2.02–2.33, *p* < 0.05) and burnout (*OR* = 2.51–3.39, *p* < 0.01) compared to the “no exposure” group. Indirect exposure alone was not related to any of the mental-health outcomes in this study.

## 4. Discussion

This study found that the type of exposure to workplace violence was related to unfavorable mental-health outcomes, over and above the effects of violence intensity. Nurses with both firsthand and secondhand exposure to workplace violence, or only firsthand, had two to four times greater odds of reporting adverse mental-health outcomes. The odds of reporting mental-health problems increased with cumulative exposure: As shown by odds ratios comparing the “direct and indirect exposure” group with their “only direct exposure” peers, nurses who were both the target of workplace violence themselves and also witnessed it occur to their co-workers were more likely to meet the criteria for mental-health problems than those who only experienced workplace violence directly. This finding is consistent with previous occupational trauma literature that suggested a dose-response relationship between the type of exposure to trauma and both mental [19,23,41,42] and physical health. In other words, nurses experiencing workplace violence both firsthand and secondhand were exposed to a higher “dose” of trauma compared to their peers who had only firsthand exposure, and were consequently more likely to meet the criteria for PTSD, anxiety, depression and burnout.

A surprising finding, however, was the non-significant relationship between “only indirect exposure” and mental-health problems. This finding is inconsistent with an extensive body of evidence that links secondhand exposure to trauma and negative mental health among various populations [4,33,34], including the nursing workforce [43,44]. Less traumatizing than direct exposure, indirect exposure to trauma is still known to deteriorate mental health [13,28,33,34,45]. The unexpected non-significant relationship between secondhand exposure to violence and adverse mental-health outcomes can partly be explained by the short-lived stress reactions triggered by this type of exposure, compared to direct exposure types [4]. In other words, it is possible that indirect exposure is not related to adverse mental-health outcomes over and above the intensity of workplace violence. The crosstab results support this possibility, as greater proportions of nurses in the “only indirect exposure” group reported mental-health problems compared to their peers with no exposure at all.

Another important finding consistent with the extant literature was the significant association between violence intensity and unfavorable mental-health outcomes. More frequent exposure to one or more types of workplace violence was related to higher odds of PTSD, anxiety, depression and burnout. This finding is consistent with an extensive body of nursing [7,46] and non-nursing research [4,47,48] that had linked poly-victimization, defined as repeated exposure to one or more types of violence over a certain time period, to poor mental health. Of note is that violence intensity is also a function of the types of violence (e.g., physical assault versus verbal threats) and their severity [49,50,51]. In this study, workplace-violence intensity was primarily a function of emotional abuse, physical assault and threat of assault, as evidenced by the high prevalence of these violence types. Herschovis and Barling found that victims of workplace aggression and mistreatment experience more adverse mental-health outcomes compared to victims of sexual harassment [49]. Other research has had different findings related to the mental-health implications of various types of workplace violence [6,27]. Given this limitation, future research should examine workplace violence using more systematic and multilevel measurement approaches that consider not only the frequency but also the types, sources and severity of workplace violence.

### 4.1. Limitations

The study’s findings should be interpreted in light of its strengths and limitations. First, to our knowledge, this was the first study to explore the impact of various types of exposure to workplace violence, including indirect and/or direct exposure, on a variety of mental-health outcomes among a provincial sample of Canadian nurses using validated scales. Despite this strength, there are also several limitations. First, similar to other research with BC nurses [6,15], this study had low response rates. However, a comparison of the overall sample (composed of nurses across a variety of roles and healthcare sectors) from which this study sample was sourced, with the provincial nursing workforce demonstrated that our sample was closely representative of the nursing population in BC with respect to age, gender and professional designation [52]. Similarly, a recent national survey by the Canadian Federation of Nurses’ Union found that proportions of nurses meeting the criteria for depression (36%) and anxiety (26%) were similar to the findings of the current study, thus providing more evidence suggesting a closely representative sample [53]. Regardless of this finding, I recommend cautiously generalizing the findings beyond the study sample. Second, it is highly likely that the voice of nurses most severely impacted by workplace violence was excluded from this study, resulting in underestimation of proportions and relationships. Third, no cause-and-effect conclusions should be made due to the cross-sectional nature of the study. Fourth, while an overwhelming majority of our sample reported exposure to multiple types of workplace violence, this study did not examine the independent effect of individual violence types (e.g., physical assault versus sexual assault) on nurses’ mental health. Finally, this study did not consider all of the personal (e.g., history of trauma, domestic violence) and workplace factors (e.g., workplace support) important to nurses’ mental health. As such, future research should study workplace violence and its implications among healthcare workers using more sophisticated methods and robust measurement techniques that comprehensively operationalize workplace violence and include personal and workplace factors important to coping with occupational stressors.

### 4.2. Implications

In this study, the type of exposure to workplace violence was found to be important to nurses’ mental health. The likelihood of adverse mental health increased with cumulative exposure such that nurses with two types of exposure were more negatively impacted compared to their peers with one type of exposure only. This finding is concerning because three-fourths of nurses in this study reported experiencing workplace violence both directly and indirectly. As workplace violence is an underreported phenomenon among nurses, this proportion is likely underestimated [54].

The study findings have some implications for practice, policy and research. First, workplace-violence prevention ought to be an urgent undertaking in healthcare. Previous research identified a series of prevention strategies important to nurses’ workplace safety and found, among them, having employers that listened to their staff was essential for nurse safety [16]. Therefore, more inclusive approaches that consider nurses as equal partners in designing and implementing practices, policies and interventions focused on violence prevention in healthcare is essential. To date, there have been commendable efforts to prevent workplace violence in healthcare such as the “zero tolerance policy” and “prevention campaigns” [16]. Despite these steps forward, more needs to be done both in terms of prevention and also for better supporting nurses who have already been impacted by chronic exposure to firsthand and/or secondhand workplace violence.

In BC, since 2012 the Workers Compensation Act was amended to more clearly describe employee compensation for work-related mental illness [55]. In 2019, various institutions and entities such as the WorkSafeBC, BC Nurses’ Union, and the provincial government joined forces to successfully expand the legislation to include the provincial nursing workforce. According to the presumptive legislation, if a nurse in BC develops a mental-health disorder after direct exposure to a traumatic incident at work such as workplace violence, the disorder will be presumed to have been caused by their work. As a result of the legislation, there will be a more-expedited claim submission and decision process followed by quicker access to mental-health resources and supports [55]. A limitation, however, is overlooking the cumulative effect of exposure type on mental health, as identified in this study, in the legislation. Nurses experiencing workplace violence directly and indirectly do not receive greater compensation or better access to resources or supports compared to their peers who have experienced violence only firsthand. Similarly, the presumptive legislation does not recognize the traumatic impact of indirect exposure to workplace violence on mental health. In other words, nurses experiencing trauma after witnessing workplace violence secondhand are not supported by the legislation. Therefore, there is room for improvement through better support and resources that include considerations of the types of exposure.

The findings also have some research implications. There is a need for more effective ways of measuring workplace violence from multiple angles and dimensions including, but not limited to, various types of exposure. Similarly, gaining a better understanding of the short- and long-term implications of workplace violence for nurses’ mental health requires more systematic and sophisticated methods such as longitudinal designs with quantitative and qualitative data.

## 5. Conclusions

This study found that the types of exposure to workplace violence were important considerations for understanding nurses’ mental health. Nurses experiencing workplace violence both firsthand and secondhand, as well as those with only first-hand exposure, were two to four times more likely to report adverse mental-health outcomes than those who reported no exposure. Currently, policies and practices do not take into account the importance of the types of exposure to workplace violence. Therefore, there is an urgent need for better nurse workplace safety through more collaborative efforts, and more effective research, policy and practices that are tailored to nurses’ needs.

## Figures and Tables

**Table 1:** Descriptive statistics on demographics and key variables (*N* = 2958).

Variable	Frequency (%)	Mean (SD) Range
Age		38.5 (11.2) (21–70 or older)
Gender		
Female	2698 (91.2)	
Male	260 (8.8)	
Professional Designation		
Licensed Practical Nurses	380 (12.8)	
Registered Nurses	2455 (83.0)	
Registered Psychiatric Nurses	123 (4.2)	
Employment status		
Full-time	1866 (63.1)	
Part-time	744 (25.2)	
Casual	347 (11.7)	
Workplace violence intensity (Sum score)		6.8 (5.5) 0–30
Physical assault	2187 (73.9)	1.4 (1.4)
Threat of assault	2455 (83.0)	2.0 (1.7)
Emotional abuse	2541 (85.9)	2.1 (1.7)
Verbal sexual harassment	1750 (59.2)	1.1 (1.3)
Sexual assault	384 (13.0)	0.2 (0.5)
Workplace violence exposure type		
No exposure	138 (4.7)	
Only-indirect exposure	52 (1.8)	
Only-direct exposure	549 (18.6)	
Both direct and indirect exposure	2219 (75.0)	
PTSD		
No (≤45)	1480 (50.3)	
Yes (>45)	1460 (49.7)	
Anxiety		
No to mild anxiety (0–9)	2103(71.9)	
Moderate to severe anxiety (10–28)	820 (28.1)	
Depression		
No or mild depression (0–9)	2013 (68.9)	
Moderate to severe depression (10–27)	909 (31.1)	
Burnout		
No	1884 (63.7)	
Yes	1074 (36.3)	

**Table 2:** Crosstab analysis of workplace violence exposure type by mental health outcomes (*N* = 2958).

	PTSD		Anxiety		Depression		Burnout	
Type of Exposure	No (%)	Yes (%)	No (%)	Yes (%)	No (%)	Yes (%)	No (%)	Yes (%)
No exposure	116 (85.3)	20 (14.7)	127 (94.8)	7 (5.2)	122 (91.0)	12 (9.0)	128 (92.8)	10 (7.2)
Only-indirect exposure	43 (82.7)	9 (17.3)	46 (88.5)	6 (11.5)	43 (82.7)	9 (17.3)	46 (88.5)	6 (11.5)
Only-direct exposure	345 (63.2)	201 (36.8)	424 (78.2)	118 (21.8)	416 (76.8)	126 (23.2)	415 (75.6)	134 (24.4)
Both direct and indirect exposure	976 (44.2)	1230 (55.8)	1506 (68.5)	689 (31.4)	1432 (65.3)	762 (34.7)	1295 (58.4)	924 (41.6)
N	1480	1460	2103	820	2013	909	1884	1074
Pearson Chi-Square	157.09 ***		64.33 ***		64.37 ***		125.10 ***	

*** *p* < 0.001.

**Table 3:** Binary logistic regression on workplace violence exposure type predicting mental health outcomes (*N* = 2958).

Variable	PTSD ^1^	Anxiety ^2^	Depression ^3^	Burnout ^4^
	Odds Ratio (95% CI)	Odds Ratio (95% CI)	Odds Ratio (95% CI)	Odds Ratio (95% CI)
Age	0.99 ** (0.98–1.0)	0.98 *** (0.97–0.99)	0.99 (0.99–1.00)	0.97 *** (0.96–0.98)
Gender ^5^	0.81 (0.61–1.06)	0.58 ** (0.42–0.80)	0.76 (0.57–1.03)	1.06 (0.80–1.41)
Professional Designation ^6^	1.10 (0.87–1.39)	1.04 (0.81–1.34)	0.90 (0.71–1.15)	1.44 ** (1.12–1.86)
Employment status ^7^	0.85 (0.66–1.08)	0.97 (0.75–1.27)	0.78 (0.60–1.02)	0.93 (0.72–1.20)
Workplace violence intensity	1.11 *** (1.10–1.13)	1.09 *** (1.08–1.11)	1.10 *** (1.08–1.12)	1.13 *** (1.11–1.15)
Only-indirect exposure	1.18 (0.50–2.82)	2.31 (0.74–7.25)	2.08 (0.82–5.27)	1.63 (0.56–4.77)
Only-direct exposure	2.18 ** (1.31–3.64)	3.49 ** (1.58–7.72)	2.02 * (1.08–3.80)	2.51 ** (1.27–4.95)
Both direct and indirect exposure	3.11 *** (1.89–5.13)	3.85 ** (1.76–8.41)	2.33 ** (1.26–4.32)	3.39 *** (1.74–6.60)
Nagelkerke R^2^	15.2%	11.3%	10.6%	19.0%

CI = confidence interval. ^1^ PTSD: below cutoff (0) versus above cutoff (1); ^2^ Anxiety: below cutoff (0) versus above cutoff (1); ^3^ Depression: below cutoff (0) versus above cutoff (1); ^4^ Burnout: below cutoff (0) versus above cutoff (1); ^5^ Gender: female (0) versus male (1); ^6^ Professional designation: LPN (0) versus registered nurse (1); ^7^ Employment status: casual (0) versus full-time or part-time (1). * *p* < 0.05, ** *p* < 0.01, *** *p* < 0.001

## Data Availability

The data presented in this study are available on request from the corresponding author. The data are not publicly available due to ethical and privacy restrictions.
